# Baseline metabolomic profile as potential biomarker for weight change after Roux-en-Y gastric bypass (RYGB) surgery

**DOI:** 10.21203/rs.3.rs-7256000/v1

**Published:** 2025-09-01

**Authors:** Vidhu Thaker, Wanjun Gu, Shuliang Deng, Nicholas Stylopoulos, Clary Clish, Joel Hirschhorn, Rany Salem

**Affiliations:** Columbia University Irving Medical Center; University of California at San Francisco; Beth Israel Deaconess Medical Center; Boston Children’s Hospital; Broad Institute of Harvard and MIT; Boston Children’s Hospital; University of California at San Diego

## Abstract

Metabolic and bariatric surgery (MBS) is the most effective intervention for sustained weight loss and cardiometabolic improvement in individuals with severe obesity. However, long-term outcomes vary, with many patients experiencing weight regain. The biological determinants of this variability remain incompletely understood. Given the integrative nature of the metabolome—capturing interactions among host genetics, diet, microbiota, and environmental exposures—we hypothesized that baseline circulating metabolites could stratify individuals into distinct long-term weight trajectory groups. We profiled untargeted fasting plasma metabolites in a nested case-control study within the Longitudinal Assessment of Bariatric Surgery (LABS-2) cohort. From these metabolites, a 13-metabolite risk score (MetRS) predictive of weight regain five years after Roux-en-Y gastric bypass was derived. The MetRS, which captures pathways including fatty acid oxidation, bile acid conjugation, and microbial-host co-metabolism, outperformed clinical variables in predicting long-term weight outcomes. Its performance was evaluated in two independent cohorts, including one assessed a median of seven years post-surgery. Genomic analyses identified common variants in loci including AGXT2 and SLC7A5 associated with key MetRS metabolites, suggesting a heritable component to the observed metabolic signature. Together, these findings lay the groundwork for a clinically actionable framework to identify individuals at risk for weight recidivism and support the integration of metabolic profiling into preoperative assessment for personalized obesity care.

Obesity and its cardiometabolic complications are a leading contributor to global disease burden. While emerging pharmacotherapies induce weight loss, their benefits often wane upon discontinuation^1^. In contrast, metabolic and bariatric surgery (MBS) is the most effective approach for sustained weight loss and obesity-related metabolic disease remission^2–4^. In 2022, approximately 280,000 MBS procedures were performed in the United States, representing 1% of the eligible population^5^. Among the available surgical options, laparoscopic sleeve gastrectomy is currently the most frequently performed procedure. In observational studies, Roux-en-Y gastric bypass (RYGB) has been associated with greater long-term weight loss and improved cardiometabolic profiles^6–8^. On average, RYGB results in a 35–40% reduction in weight, with 70–80% of individuals maintaining this loss ≥ 5 years postoperatively^8^. Despite these benefits, outcomes vary, with some patients regaining weight to preoperative levels^9–12^. Predictive factors such as younger age^13^, white race^14^, preoperative weight loss^15–17^, behavioral characteristics^18^, and select genetic variants^12,19^, have been proposed, but no clinically validated biomarkers currently exist to forecast long-term success following MBS.

The human metabolome captures the dynamic interplay of host genetics, diet, pharmacologic exposures, and gut microbial activity. Untargeted metabolomics enables comprehensive profiling of both known and unannotated metabolites, facilitating biomarker discovery and mechanistic insight into obesity-related metabolic dysfunction^20,21^. Specific metabolite signatures—including branched-chain amino acids (BCAAs), lipid species, and intermediates of central carbon metabolism—have been linked to obesity, insulin resistance, and type 2 diabetes^21^. Notably, BCAAs and related metabolites show distinct trajectories following MBS compared to medical weight loss, suggesting surgery-specific metabolic remodeling^22–24^. However, prior studies have often lacked long-term follow-up, sufficient statistical power, or broad metabolite coverage.

We hypothesized that untargeted metabolomics could identify metabolites predicting long-term weight outcomes following bariatric surgery. To test this, we profiled pre-surgery fasting plasma from a subset of individuals who underwent RYGB in the Longitudinal Assessment of Bariatric Surgery (LABS-2) study^25–27^. Using a nested case–control design, we compared individuals with sustained weight loss to those with substantial weight regain. We then evaluated the relationship of the prioritized metabolites to obesity phenotype in two external datasets: a long-term post-RYGB clinical cohort assessed approximately seven years after surgery^28^, and a population-based cohort representing extremes of BMI^29^. Finally, genome-wide association analyses in an independent cohort were used to examine the heritable basis of key metabolites associated with long-term surgical outcomes.

To identify clinically meaningful subgroups, we applied latent class growth mixture modeling (LCGMM) to 7-year excess body weight (EBW) trajectories from 1,590 LABS-2 participants. EBW was defined as the measured weight minus ideal body weight corresponding to BMI of 25 kg/m^2^. Three latent trajectory classes emerged: Class 1 (n = 100, 6.3%) showed modest initial weight loss followed by regain; Class 2 (n = 108, 6.8%) achieved and maintained sustained weight loss; and Class 3 (n = 1,382, 86.9%) had lower baseline EBW and modest, sustained weight loss. Baseline EBW was comparable between Classes 1 and 2 (101.0 ± 19.2 kg vs. 105.5 ± 17.8 kg, p = 0.09), but significantly lower in Class 3 (58.1 ± 15.5 kg, p < 0.001). By year five, weight divergence became pronounced: Class 1 regained 16.1 ± 12.9 kg, Class 2 continued to lose weight (–14.0 ± 12.9 kg), and Class 3 experienced partial regain (6.5 ± 10.8 kg) (Fig. 1a and Extended Data Fig. 1). Given its distinct baseline profile and non-overlapping weight dynamics, Class 3 was excluded from subsequent analyses.

To focus on predictors of divergent outcomes, we selected 80 individuals from the sustained weight loss group (Class 2) and matched 1:1 to individuals from the weight regain group (Class 1) using propensity scores based on baseline BMI, age, sex, race, and renal function (Fig. 1b and Extended Data Fig. 2). Baseline clinical and biochemical measures—including fasting glucose, insulin, lipids, ghrelin, leptin, and inflammatory markers—were similar between groups (Extended Data Table 1). Weight trajectories were not influenced by sex or surgical center (Extended Data Fig. 3).

To capture the non-linear dynamics of weight change, we modeled EBW using a broken-stick linear mixed-effects model with knots at 6, 12, and 24 months. This spline-based approach substantially improved model fit relative to linear regression (R^2^ = 0.98 vs. 0.58, Figure 1 c–e), enabling interpolation across follow-up visits and consistent weight trajectory estimation. At year five, model-derived mean EBW was 72.4 ± 15.2 kg (69.8% of baseline) in the weight regain group compared to 29.1 ± 20.8 kg (27.0% of baseline) in the sustained weight loss group.

Untargeted metabolomics was performed on preoperative fasting plasma using liquid chromatography–mass spectrometry (LC-MS) across four acquisition modes: HILIC positive and negative, C18 negative, and C8 positive. This profiling enabled detection of amines and polar metabolites, central carbon intermediates, free fatty acids, and lipids. Of 786 initially detected features, 591 unique compounds remained after collapsing redundancies across modes. Following QC exclusions—missingness in > 25% samples (n=39), coefficient of variation > 20% in pooled QC samples (n=10), and batch effects (n=1)— a final set of 541 high-confidence metabolites were retained. No samples failed QC. Data were log-transformed, adjusted for covariates, and inverse-normal transformed. Missing values were imputed using multiple imputation by chained equations (MICE) to preserve cohort structure and variance^30^. This preprocessing pipeline was applied consistently across all cohorts.

Percent excess body weight at year five (%EBW5) was defined as the proportion of baseline EBW remaining five years after RYGB (Figure 1f). Higher %EBW5 reflects reduced surgical efficacy, whether due to suboptimal initial weight loss or subsequent weight regain. In linear regression models, 62 metabolites were nominally associated with %EBW5 (*p* < 0.05; Extended data Fig. 4), with five meeting a false discovery rate (FDR) threshold (q < 0.05): 3-methyladipate/pimelate, glucuronate, dimethylguanidino valeric acid (DMGV), 13-hydroxyoctadecadienoic acid (13-HODE), and b-aminoisobutyric acid (BAIBA). Twelve metabolites, including kynurenine, bilirubin, α-carboxyethyl hydrochroman (CEHC), leucine, tryptophan, GABA, and homoarginine, were positively associated with %EBW5, indicating higher levels in individuals with poorer surgical outcomes. In contrast, 50 metabolites showed inverse associations, including seven triacylglycerols, three lysophosphatidylcholines, four phosphatidylcholines, and two lysophosphatidylethanolamines. These inverse associations suggest a favorable metabolic profile associated with sustained weight loss. Collectively, these metabolites implicate biologically interconnected pathways – such as fatty acid oxidation, inflammation, bile acid metabolism, and gut-microbiome-derived xenobiotic processing – as markers of long-term variability following MBS.

To prioritize predictive features, we applied bidirectional stepwise linear regression to the 62 nominally associated metabolites, resulting in a 13-metabolite panel (five positively and eight negatively associated with weight regain; Fig 2 a–c). Although DMGV met FDR significance, it was excluded from the final model due to its strong correlation with BAIBA (Pearson’s *r* = −0.56, *p* < 10^−15^), exceeding a prespecified correlation threshold of |r| > 0.5. A Metabolite Risk Score (MetRS) was constructed as a weighted sum of standardized metabolite concentrations with weights derived from the corresponding regression coefficients.

To reduce overfitting and ensure robustness of the MetRS, candidate features were limited to metabolites nominally associated with %EBW5 and AIC-based feature selection by stepwise regression. The resulting model demonstrated strong internal validity, with consistent performance across 100 repetitions of 10-fold cross-validation (root mean squared error [RMSE] = 21.45 ± 1.32; mean absolute error [MAE] = 17.24 ± 1.10, Fig 3 c). Residual diagnostics showed no major violations of linearity, homoscedasticity, or normality assumptions, and variance inflation factors (VIFs) confirmed acceptable multicollinearity. These findings support the internal consistency of the risk score and its suitability for further testing in external datasets.

As expected, MetRS values were higher in individuals with weight regain compared to those with sustained weight loss (*p* < 0.001; Fig. 3a). To determine whether the MetRS was predictive beyond standard clinical variables, we first evaluated factors independently associated with weight regain. In conditional logistic regression models, higher baseline EBW (OR 1.03, 95% CI: 1.01–1.05, *p* = 0.01) and younger age (OR 0.95, 95% CI: 0.91–0.99, *p* = 0.02) were significantly associated with increased risk of weight regain. Incorporation of the MetRS into the model substantially improved the discriminative performance, increasing the area under the receiver operating characteristic curve (AUC) from 0.60 to 0.75 (*p* < 0.001, Fig. 3 b). The MetRS remained a strong predictor of weight regain in both unadjusted and multivariable-adjusted models (OR 2.63, 95% CI: 1.60, 4.31, *p* = 0.000132; Fig. 3 d), supporting its potential as a molecular marker for long-term surgical response.

To assess the reproducibility of the MetRS in an independent post-surgical context, we applied a modified version of the score—including 10 of the original 13 metabolites—to a cohort evaluated a median of 7.2 years after RYGB (n=35, Extended Data Fig. 5). MetRS values were significantly higher in individuals with documented weight regain (Fig. 4a, p=0.01) and correlated with the magnitude of regain (Pearson’s r = 0.56, p < 0.001). Each standard deviation increase in MetRS was associated with a 12.93% greater weight regain (95% CI: 7.01–18.85, *p* < 0.001, Fig. 4 c), independent of baseline clinical characteristics.

To evaluate whether the MetRS reflects broader features of adiposity beyond surgical settings, we next examined its association with obesity status in a population-based cohort from the Estonia Biobank (n=198; Extended Data Fig. 6). A modified version of the score, incorporating 8 MetRS components, was significantly elevated in individuals with obesity (BMI 40.5 ± 4.8 kg/m^2^) compared to lean controls (BMI 18.0 ± 1.1 kg/m^2^, *p* = 0.01, Fig. 4 b). In linear regression models, MetRS was modestly associated with BMI (0.10 kg/m^2^ per SD increase in MetRS, 95% CI: 0.00–0.20, *p* = 0.05), suggesting that the score may capture general features of metabolic status relevant to both surgical and non-surgical populations.

These findings suggest that MetRS captures a stable metabolic signature that persists after surgery. Its reproducibility in an independent post-RYGB cohort implies that it may reflect intrinsic traits—such as metabolic flexibility or resilience—programmed prior to intervention. The consistency of this signature across individuals and over time supports the concept of a metabolic set point, potentially influenced by genetic factors. Identifying such genetic contributions may help disentangle causes from acquired metabolic features, improve interpretability, and enhance risk stratification in clinical care. To investigate this, we next assessed whether the MetRS and its components are influenced by common genetic variation.

We performed genome-wide association studies (GWAS) in the Framingham Heart Study (FHS) Offspring cohort to identify genetic determinants of the MetRS and its component metabolites (Extended Data Fig. 8A). The MetRS and five components—glucuronate, hippurate, kynurenine, BAIBA, and *N*-carbamoyl-β-alanine—were each associated with genome-wide significant loci (*p* < 5×10^−8^, Extended Data Fig. 8B)). At the *AGXT2* locus, rs13174300 was associated with the MetRS and remained significant after conditioning on nearby variants, indicating an independent contribution to the composite score. In contrast, three distinct *AGXT2* variants—rs37376, rs163910, rs11749934—were independently associated with BAIBA (Extended Data Fig. 8C), suggesting multiple, non-redundant genetic signals at this locus regulating BAIBA levels. These findings underscore the polygenic nature of AGXT2 and raise the possibility that rs13174300 may act through pathways beyond BAIBA.

Additional genome-wide significant loci were identified for individual metabolites, including rs9594030 near *LINC00351* (glucuronate), rs9926829 in *SLC7A5* (kynurenine), rs117638208 (intergenic; hippurate), and rs182678014 (*N*-carbamoyl-β-alanine). Notably, the association between *SLC7A5* and kynurenine has been previously reported in metabolite-QTL studies^31,32^, whereas the other loci appear to be novel, representing independent genetic signals not previously reported. Collectively, these associations highlight a broader network of enzymatic and transporter genes in the regulation of metabolic traits that may be influence long-term outcomes following bariatric surgery.

The pre-surgical metabolites associated with long-term post-RYGB weight outcomes spanned domains of lipid metabolism, amino acid catabolism, xenobiotic clearance, and host-microbial interactions. 3-methyladipate, a dicarboxylic acid produced via ω-oxidation^33,34^ and 13-HODE, a lipid-derived mediator from linoleic acid^35,36^, were both reduced in individuals with weight regain. These findings may reflect altered lipid handling or oxidative balance, though additional evidence is needed to implicate broader disruptions in fatty acid metabolism. Glucuronate, a metabolite involved in hepatic conjugation and bile acid turnover^37,38^, was also lower in weight regain group, raising the possibility of altered enterohepatic or GLP-1 related signaling^39,40^. Additionally, 4-hydroxyhippuric acid, a gut microbiome -derived polyphenol metabolite, was elevated among those with sustained weight loss. While this metabolite has been linked to higher fruit and vegetable intake and improved metabolic profile^41,42^, its elevation may reflect differences in dietary exposure, microbial activity, or both. These associations warrant further investigation to determine whether they represent causal mechanisms or downstream consequences of divergent weight trajectories.

Other metabolites in the MetRS were related to nucleotide metabolism and amino acid catabolism. Hydroxyproline, a byproduct of collagen turnover^43^, along with nucleoside derivatives 1-methylguanosine^44^ and N-carbamoyl-β-alanine^45^, were enriched in individuals with sustained weight loss, suggesting preserved redox status and mitochondrial integrity. Lower preoperative levels of N-acetylserine—a serine pathway intermediate involved in one-carbon metabolism and antioxidant defense^46^—may reflect impaired serine-dependent metabolic flexibility. Given the role of serine in glucose regulation, insulin sensitivity^47^, and hypothalamic energy sensing^48,49^, diminished N-acetylserine may serve as a marker of systemic metabolic stress that predisposes individuals to weight regain following MBS. Conversely, β-aminoisobutyric acid (BAIBA)—a myokine-like molecule linked to fatty acid oxidation and insulin sensitivity^50,51^—was unexpectedly associated with adverse outcomes. Although BAIBA has been shown to promote thermogenesis and improve glucose handling, its elevation here may reflect a compensatory response to early mitochondrial dysfunction or a maladaptive signal in this surgical context. Supporting this, common variants in AGXT2, the BAIBA-regulating enzyme^52^, were associated with both circulating BAIBA levels and the composite MetRS. This may reflect either a strong contribution of BAIBA to the overall MetRS or a genetic signal for BAIBA that remains detectable even within the composite model.

Additional metabolites associated with weight regain included kynurenine, a tryptophan-derived metabolite linked to inflammation and appetite regulation via aryl hydrocarbon receptor signaling^53^; homoarginine, linked to nitric oxide metabolism^54^; and malonate, a short-chain dicarboxylate that modulates hypothalamic nutrient sensing^55^. Collectively, these associations point to perturbations in inflammatory signaling, vascular tone, and central energy regulation as potential contributors to poor long-term surgical outcomes.

Most of the identified metabolites have not been previously linked to obesity or post-bariatric outcomes, underscoring the novelty of this metabolomic signature. The 13-metabolite MetRS was consistently associated with long-term weight regain and demonstrated predictive utility across two external cohorts with differing clinical contexts. Genetic association analyses in the FHS further linked several MetRS components to common variants in genes such as *AGXT2*, *SLC7A5*, and *LINC00351*, suggesting a potential heritable contribution to metabolic features captured by the score. Although causal inference was beyond the scope of this study, future application of Mendelian Randomization in post-MBS cohorts may help clarify the directionality of these associations.

Taken together, these findings suggest that the MetRS reflects a multifactorial metabolic phenotype integrating signals from diverse biochemical domains. While certain individual associations—such as the elevated BAIBA—raise the possibility of early metabolic strain in individuals predisposed to weight regain, additional pathway-level analyses will be needed to assess whether these signals converge on broader physiological mechanisms. Overall, these data support further investigation of metabolomic risk profiling as a tool to better understand inter-individual variability in long-term surgical outcomes.

This study leverages a deeply phenotyped, longitudinal cohort, and applies rigorous untargeted metabolomics for the biomarker discovery, with subsequent evaluation in independent datasets. Several limitations merit consideration. First, the discovery sample size was modest, which may limit detection of weaker associations and carries a risk of false-positive findings, though the consistent performance of the MetRS in an independent cohort provides some reassurance. Second, most participants were of European ancestry, and future studies are needed to assess generalizability across ancestries. Third, while the models were adjusted for key clinical variables, residual confounding from unmeasured factors—such as diet, physical activity, or medication use—cannot be excluded. Fourth, the absence of concurrent gut microbiome and bile acid profiling limits mechanistic insight into host–microbiome interactions. Fifth, causal inference cannot be drawn from metabolite associations alone, and it remains unclear whether the identified metabolites drive, reflect, or buffer weight change. Finally, while the MetRS was tested in a post-surgical cohort, the lack of preoperative samples in that setting precludes temporal resolution of its dynamics.

Despite these limitations, our findings suggest that preoperative metabolomic profiling may improve risk stratification beyond traditional clinical predictors. The implicated pathways—including fatty acid oxidation, redox and one-carbon metabolism, and microbial xenobiotic processing—are biologically plausible and in some cases modifiable via diet, microbiome-targeted therapies, or metabolic interventions. The association of MetRS components with genetic loci further underscores a heritable component to metabolic resilience after surgery.

These findings advance the concept of precision bariatrics: tailoring perioperative management and long-term follow-up based on individualized molecular risk. To realize this vision, further validation of metabolite-based risk scores in larger and more varied populations is essential, alongside mechanistic studies to clarify the underlying biological pathways. In clinical practice, implementation could begin with a preoperative screening algorithm incorporating the MetRS, derived from a fasting blood sample obtained during routine evaluation. High-risk individuals may benefit from intensified perioperative counseling, closer post-operative surveillance, and early behavioral or pharmacologic support. As genomic studies of weight regain emerge, integrating genetic data could further refine risk prediction—especially in younger patients or clinically ambiguous cases. Finally, development of a focused clinical assay targeting validated metabolites could support broader use of this strategy across bariatric centers. Together, these steps outline a scalable framework for integrating metabolic profiling into surgical risk stratification and advancing personalized obesity care.

## Methods

### Study Design and Cohorts

This study integrates data from four cohorts to evaluate, validate and assess the genetic association of metabolomic predictors of weight loss response following bariatric surgery.

### Discovery cohort: Longitudinal Assessment of Bariatric Surgery (LABS-2)

LABS-2 is a multicenter, prospective observational study of adults undergoing first-time metabolic and bariatric surgery (MBS). Between 2006 and 2009, 2,458 participants (aged ≥18 years) were enrolled across 10 U.S. clinical centers^25–27^. Clinical data and biospecimens were collected at baseline (≤30 days before surgery), 1-year post-surgery, and annually up to 7 years. Height and weight were measured using standardized protocols. Body mass index (BMI) was calculated as weight (kg)/height (m^2^). Excess body weight (EBW) was defined as measured weight minus ideal body weight, where ideal body weight was defined corresponded to a BMI of 25 kg/m^2^.

### Participant Selection and Case-Control Design

Among the 1,539 participants with complete 7-year weight data and baseline plasma samples, a nested case–control study was conducted. Weight trajectories were modeled using latent class growth mixture modeling (LCGMM) in Mplus v7.4 to identify subgroups based on EBW trends over time. Individuals in the “sustained weight loss group” (n=80) were defined as those who maintained ≥ 50% EBW loss at year 5 post-surgery. These individuals were matched 1:1 using propensity scores to a “weight regain” group (n=80), comprising individuals who initially lost weight but subsequently regained ≥ 20% of their maximum weight lost. Matching was performed on baseline age, sex, baseline BMI, and renal function.

### Post-RYGB Clinical Dataset

This prospective cohort, recruited at Brigham and Women’s Hospital (Boston, MA, USA), included individuals who had undergone Roux-en-Y gastric bypass (RYGB) at least five years prior and were evaluated 5–10 years postoperatively^28^. Participants with weight regain—defined as ≥ 20% regain of maximum weight loss, calculated as [(current weight – nadir weight)/(initial weight – nadir weight)]—were identified (n=21) and matched to individuals with sustained weight loss (n=14) based on age, sex, preoperative BMI and comorbidity burden to minimize confounding by baseline clinical characteristics.

Exclusion criteria included active psychiatric illness, major anatomical complications, and current use of anti-obesity medications. After an overnight fast, blood samples were collected and processed for metabolomic profiling. Samples were collected at a median of 7–9 years after surgery, offering a unique opportunity to examine durable metabolic differences associated with divergent long-term surgical outcomes.

### Estonian Biobank Obesity Extremes Dataset

This external dataset was derived from the Estonian Biobank at the University of Tartu and included 200 individuals from the extremes of the BMI distribution: 100 lean (BMI < 20 kg/m^2^) and 100 with obesity (BMI > 34 kg/m^2^), matched by age, sex, and fasting duration (≥4 hours)^29^. Individuals with pregnancy, anorexia, or known wasting illnesses were excluded.

### Framingham Heart Study (FHS) Offspring Cohort

Data for the Framingham Heart Study (FHS), a prospective cohort study of CVD risk factors, was obtained through the NCBI database of Genotypes and Phenotypes (dbGaP, accession # phs000007) and utilized for GWAS analyses of associated metabolites. Metabolite profiling was performed on 2,016 fasting plasma samples from the FHS Offspring Cohort collected at Exam 5 (1991–1995) using LC-MS[32]. Metabolite data was limited to set with available genomic data, resulting in a total of 1,613 samples.

### Metabolite Profiling

Pre-surgery baseline fasting LABS-2 plasma samples were processed within 2 hours of collection and stored at −80 °C. LC-MS (Q Exactive; Thermo Scientific) was used to profile metabolites across four chemical modes: a) Amines and polar metabolites (e.g., amino acids, dipeptides); b) Central carbon metabolites (e.g., sugars, sugar phosphates, organic acids); c) Free fatty acids and intermediates (e.g., bile acids); and d) Lipids (e.g., phospholipids, sphingomyelins, glycerides).

Samples were randomized and interspersed with pooled QC samples. Progenesis CoMet v2.0 was used for peak detection and integration; metabolite identities were verified using TraceFinder v3.1 against authentic standards. Metabolites were excluded if they showed >25% missingness, coefficient of variation >20%, or missingness correlated with batch. Metabolite profiling for both the discovery (LABS-2) and the independent external cohorts was conducted using the four-mode LC-MS platform at the Broad Institute Metabolomics Platform enabling quantification of 370–560 metabolites.

### Statistical Analysis

Prior to analysis, metabolite concentrations were log-transformed and adjusted for relevant covariates, including age, sex, race, fasting time, baseline BMI, diabetes, and smoking. Missing values were imputed using the MICE package (v3.1) with three separate imputations; the median value across imputations was used to preserve data integrity while minimizing stochastic variability. To ensure normality, residuals were transformed using a rank-based inverse normal transformation. Heatmaps were generated using MetaboAnalyst v5.0. All statistical analyses were conducted using R v4.0.3. This preprocessing and normalization strategy was applied uniformly across all cohorts to ensure methodological consistency and comparability of results.

### Weight Change Trajectory Modeling

Non-linear trajectories of EBW were modeled using the broken stick model implemented in brokenstick R package (v2.0.0), which implements linear B-splines with knots placed at 6, 12, and 24 months. The model-generated EBW predictions were compared to the observed weight data to assess fit. These predicted EBW values were used to calculate percent change from baseline at five years post-surgery. Linear regression was then applied to test associations between metabolite residuals and 5-year EBW change.

### Metabolite Risk Score (MetRS)

All metabolites nominally associated with five-year EBW change (p < 0.05) were entered into a bidirectional stepwise linear regression, with model selection guided by Akaike Information Criterion (AIC) to balance model fit and parsimony. Adjusted R^2^ was used post hoc to assess explanatory power. Model performance was evaluated using repeated 10-fold cross validation (100 iterations) and bootstrap resampling to assess the stability of variable selection. Sensitivity analyses using LASSO regression were conducted to confirm the robustness of selected features. Biological relevance of the final metabolite panel was assessed through targeted literature review.

MetRS was computed for each participant as the weight linear combination of metabolites:

$$\text{MetRS}=\sum_{i=1}^{n}Z_{i}\beta_{i}$$

where b_i_ is the effect estimate and z_i_ the metabolite z-score. Conditional logistic regression was used to evaluate the predictive performance of MetRS in conjunction with clinical covariates (age, sex, baseline BMI) with model performance assessed through repeated 10-fold cross-validation. Discriminatory ability was quantified using the c-statistic. External evaluation of the MetRS was performed in the Obesity Extremes and Post-Bariatric Surgery cohorts. MetRS scores were computed using discovery-model coefficients and compared between outcome groups using t-tests.

### Framingham Heart Study (FHS) Offspring cohort

Metabolite Profiling and Processing: Data for the Framingham Heart Study (FHS) cohort^56^, a prospective cohort study of CVD risk factors, were obtained through the NCBI database of Genotypes and Phenotypes (dbGaP)^57^, with accession number phs000007. Metabolite profiling was performed on 2,016 fasting plasma samples from the FHS Offspring Cohort collected at Exam 5 (1991–1995) using liquid chromatography-mass spectrometry (LC-MS)^58^. Metabolite quality control and standardized was performed as for the other cohorts. Metabolite profiling data was filtered to set with available genetic data, resulting in a total of 1,613 samples.

### Genome-Wide Association Study of Metabolites

#### GWAS Quality control & Imputation:

A stringent quality control protocol was applied to the FHS genome-wide genotyping data retrieved from dbGaP. The protocol includes removal of subjects with discordant genetics and reported sex, samples that have > 2% missing SNPs, SNPs that have minor allele frequencies < 1%, SNPs that have > 5% missing rate, and samples that have extreme heterozygosity (± 4 SD). The SNP annotations for chromosome and base-pair positions were set to the coordinates of hg19 (GRCh37) via the liftOver tool^59^. Pairwise IBD/IBS were calculated and individuals that have excessive matching with other individuals were removed. Principal components analysis was performed with SMARTPCA^60^ and each study was projected onto 1000Genomes space. Samples that did not cluster with the expected HapMap European population were removed. SNPs that had excessive plate effects (p < 1 × 10^−7^) or excessive deviation from Hardy-Weinberg equilibrium (p < 1 × 10^−7^) were dropped. The QC protocol was performed with PLINK (v1.07)^61^ and custom R and PERL scripts. Genotypes were phased with SHAPEIT2 (v2.644)^62^ and imputed with IMPUTE2 (v2.3)^63^. FHS was imputed using 1000 Genomes phase 1 v3 cosmopolitan reference panel^64^. The imputation panel consists of approximately 22 million variants (SNPs, insertions, and deletions).

#### GWAS analysis:

Genome-wide association study (GWAS) analyses were performed in the FHS Offspring cohort to identify genetic loci associated with MetRS and constituent individual metabolites. Analyses were performed using SAIGE^65^ on autosomal chromosomes, which implements a linear mixed model that is well suited to deal for GWAS analyses of studies with samples relatedness. For X chromosome, analyses were performed using PLINK 2.0 on unrelated subsets of the FHS sample. GWAS Analyses were performed on the MRS and constituent individual metabolites, adjusted for sex, age, body mass index (BMI), and ten principal components from EIGENSOFT^60^ as covariates. To identify independent genome-wide significant loci, GWAS results were clumped using PLINK 1.9 with a genomic window size of 500kb and a linkage disequilibrium R2 cutoff of 0.1. A p value less than 5E-8 was used to establish genome-wide significance. For genetic regions exerting more than one distinctive genome-wide significant signals, conditional analysis was performed recursively conducted by controlling for the top signal in the region until only one independent signal was found. Manhattan and QQ plots were generated in R, and regional association plots were generated for genome-wide significant loci using locuszoom. Functional annotation was performed on loci reaching the genome-wide significance (P ≤ 5e^−8^) using BioThings^66^.

## Supplementary Material

This is a list of supplementary files associated with this preprint. Click to download.

- Extendeddata29Jul25.docx

## Figures and Tables

**Figure 1:**
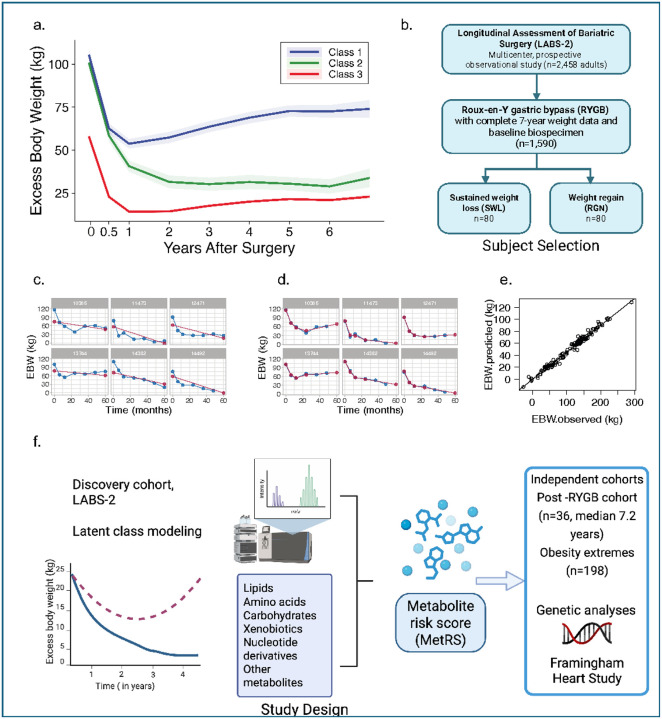
Weight trajectory modeling, subject selection, and study design. **a.** Mean EBW (kg) over 7 years post-surgery is shown for each latent trajectory group identified via latent class growth mixture modeling. EBW was defined as the difference between actual weight and ideal body weight (based on BMI 25 kg/m^2^). **Class 1 (blue):**Individuals with initial weight loss followed by substantial long-term regain. **Class 2 (green):** Individuals with sustained long-term weight loss. **Class 3 (red):** Individuals with limited weight loss and early plateau. Shaded regions represent ±2 standard errors from the mean at each time point. **b.** Flowchart of participant selection from the LABS-2 cohort, a multicenter prospective study of adults undergoing Roux-en-Y gastric bypass (RYGB), restricted to 1,590 individuals with complete 7-year weight data and preoperative biospecimens. A nested case-control design was used to identify individuals with sustained weight loss (n = 80) and those with weight regain (n = 80). **c-d.** Broken-stick linear mixed-effects model fitted to the EBW data with knots at 6, 12, and 24 months, enabling interpolation across time points and estimating individual-specific weight trajectories over 7 years. **e.** Correlation plot of observed and predicted EBW from the broken-stick linear mixed-effects model. **f.** Overview of study workflow. Untargeted metabolomics was performed on fasting plasma collected preoperatively in the LABS-2 cohort. A 13-metabolite risk score (MetRS) was derived in the discovery cohort and validated in two independent datasets: a post-RYGB clinical cohort (*n*= 36, median 7.2 years post-op) and an obesity extremes cohort from the Estonian Biobank (*n* = 198). Genetic associations with key metabolites were assessed in the Framingham Heart Study.

**Figure 2:**
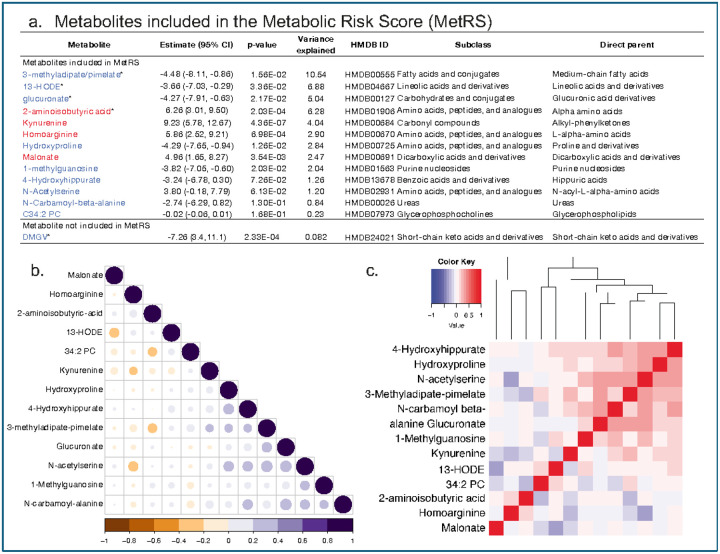
Metabolite Risk Score (MetRS). **a.** Metabolites included in the MetRS, **b.**Bubble plot depicting Pearson correlation coefficients between metabolites included in the MetRS. The size and color of each circle represent the strength and direction of the correlation, with blue indicating positive and red indicating negative correlations. Darker and larger circles reflect stronger correlations. Only the lower triangle of the correlation matrix is shown to avoid redundancy. The color scale bar on the right ranges from −1 (strong negative correlation) to +1 (strong positive correlation), **c.**Hierarchical clustering heatmap depicting pairwise Pearson correlation coefficients among the 13 metabolites comprising the MetRS. Metabolites are ordered by unsupervised hierarchical clustering based on correlation similarity. The heatmap scale ranges from strong negative correlations (dark blue) to strong positive correlations (dark red). The diagonal reflects perfect self-correlation. This analysis highlights independent and co-regulated features contributing to the composite MetRS.

**Figure 3:**
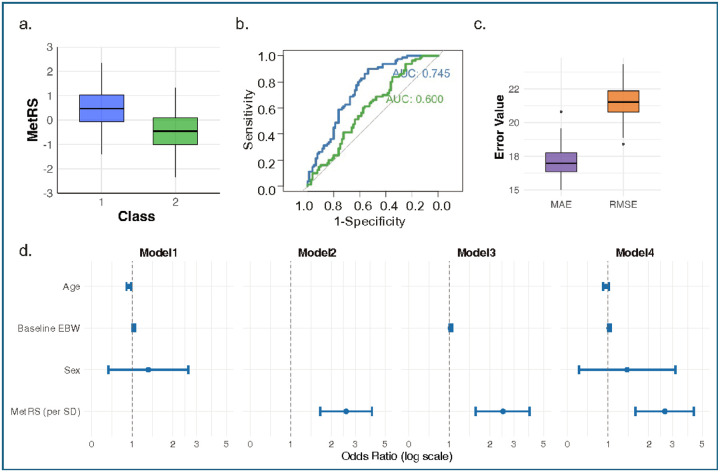
Metabolite Risk Score (MetRS) performance in the LABS-2 cohort. **a.** Boxplot of MetRS in the Class 1 (weight regain) and Class 2 (sustained weight loss) groups, **b.** Receiver Operating Characteristic (ROC) curves compares the performance of logistic regression models with and without the MetRS for predicting weight regain after RYGB. The blue curve represents the model including baseline excess body weight (EBW), age, sex and MetRS (AUC =0.745), showing improved discrimination over the green curve representing the model without MetRS (AUC = 0.600). **c.** Cross-validation performance metrics for the metabolite-based predictive model. Boxplots summarize the distribution of error metrics from 100 iterations of 10-fold cross-validation used to evaluate model performance. RMSE (Root Mean Squared Error) reflects the average magnitude of prediction errors, weighted by larger deviations. MAE (Mean Absolute Error) captures the average absolute difference between predicted and observed values, **d.** Forest plots summarizing odds ratio (OR) and 95% confidence intervals for predictors of weight regain across four logistic regression models. Model 1 includes baseline EBW, age, and sex; Model 2 includes MetRS only; Model 3 includes MetRS and Baseline EBW, and Model 4 includes baseline EBW, age, sex and MetRS.

**Figure 4:**
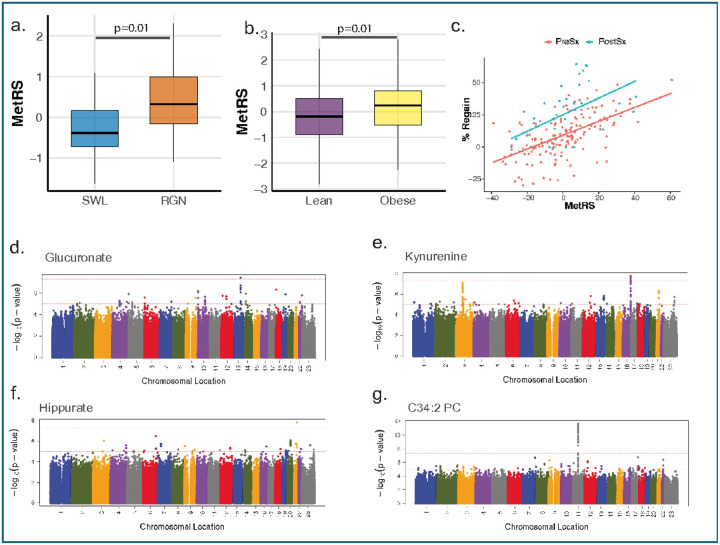
Evaluation of the Metabolic Risk Score (MetRS) in external cohorts. **a.** Mean MetRS values in lean and obese participants from Estonia Biobank Obesity Extremes cohort (n=198), **b.** MetRS in an independent post-RYGB clinical cohort (n=35) comparing individuals with long-term weight regain (RGN) versus those with sustained weight loss (SWL), **c.**Correlation of MetRS with percentage weight regain (%regain) with the MetRS in the pre-surgical metabolite profiling (red) and post-surgical profiling (blue). Manhattan plots for the genome wide association study of the metabolites in the Framingham Heart Study cohort (n = 1508) showing genome wide association for **d.**glucuronate, **e.** Kynurenine, **f.** Hippurate, and **g.** C34:2 Phosphatidyl choline.

## Data Availability

Summary-level data and code supporting the findings are available upon request from the corresponding authors. Genotypic and phenotypic data from the Framingham Heart Study are publicly available via dbGaP (accession: phs000007).
